# Assessment of correlation between asthenozoospermia and mitochondrial DNA mutations in Egyptian infertile men

**DOI:** 10.1186/s43141-020-00111-0

**Published:** 2021-01-18

**Authors:** Mohamed M. Abd Elrahman, Aida I. El makawy, Mohamed S. Hassanane, Sally S. Alam, Nagwa H. A. Hassan, Medhat K. Amer

**Affiliations:** 1 Cell Biology Dept. , Division of Genetic Engineering and Biotechnology Research, National Research Centre, 33 El Bohouth St., Dokki, P.O.12622, Giza, Egypt; 2 Zoology Dept., Faculty of Science, Ain Shams University, Cairo, Egypt; 3 Surgery Andrology and infertility Department, Faculty of Medicine, Cairo University, Cairo, Egypt

**Keywords:** Male fertility, mtDNA, Mutations, Sperm motility, ROS, Lipid peroxidation

## Abstract

**Background:**

Asthenozoospermia is a chief reason for male seminal pathologies with an impression of around 19% of infertile patients. Spermatozoa mitochondrial DNA variations seem to link with low sperm motility. The objective of the study was to assess the relation between mitochondrial mutations and male sterility, especially in asthenozoospermia. The patient semen samples were investigated by studying the sperm physical characters; motility, viability, and morphological parameters were then classified into normozoospermia and asthenozoospermia. In addition, the level of malondialdehyde (MDA) as a bio-indicator of lipid peroxidation, seminal fructose, and total antioxidant capacity (TAC) were estimated. For molecular analysis, DNA from the semen samples was extracted using a DNA extraction kit. ND1, ND2, and ATPase6 genes were amplified by using a specific primer. After the purification procedure, each PCR product was sequenced to identify the single nucleotide polymorphisms (SNPs) in selected genes.

**Results:**

A significant negative correlation between seminal plasma malondialdehyde levels and sperm motility was detected. Meanwhile, TAC analysis revealed significantly lower activity (*p* ≤ 0.05) in the sample of asthenozoospermic than in normozoospermic men. As regards the seminal plasma fructose, there was no significant difference in the fructose level of normozoospermia and asthenozoospermia cases. At the molecular level, 31 diverse nucleotide substitutions were recognized in mitochondrial DNA. Only ten (10) mutations led to amino acid transformation: four have deleterious effects, four are benign, and the other two have conflicting effectiveness.

**Conclusions:**

This study is the first in Egypt that is concerned with studying the relationship between the mitochondrial DNA mutations in human spermatozoa of asthenozoospermic patients and fertility. The results displayed scientific indications evidenced that there is an association between mitochondrial mutations and male infertility.

## Background

Male infertility denotes male’s incapability to cause fertile female pregnancy. It affects around 7% of men all over the world [1]. Male sterility is generally owed to semen shortages, and semen integrity is deemed as a replacement measure of fecundity [2]. The utmost mutual reason for male infertility is idiopathic. Oxidative stress would show an energetic character in the etiology of male infertility as a result of its targeting to spermatozoa plasma membrane a polyunsaturated fatty acid [3].

Sperm motility is an imperative determinant of fertility, where it is a public feature of flagellate spermatozoa and is an important sperm characteristic for the process of fertilization [4]. Asthenozoospermia is a chief reason for male seminal pathologies that impact around 19% of infertile patients. It is characterized by decreasing progressively motile percentage (PR) spermatozoa under 32% [5]. Spermatozoa motility is very reliant on numerous metabolic pathways and regulatory mechanisms. Further, the specific gene defect association and any deformities of these factors could be accountable for poor sperm motility and accordingly sterility [6]. Motility is an energetic trait of spermatozoa for positive fertilization that is essential for passage through the female genital tract to penetrate the oocyte zona pellucida. Sperm motility is the origin of the sperm tail flagella movement by ATP-derived energy, formed in the mid-piece positioned mitochondrion [7].

Human mitochondria perform a crucial role in energy manufacture for all organisms through the synthesis of adenosine triphosphate (ATP) in oxidative phosphorylation. Sperm mitochondria are an industrial unit for energy production via oxidative phosphorylation (OXPHOS), viz ATP synthesis, and they play crucial roles in spermatogenesis, differentiation, and functioning of germ cells [8]. The spermatozoon of a mammal contains between 22 and 75 mitochondria situated in the intermediate piece of the flagellum, ensuring correct flagellar function and sperm motility that deem as a vital parameter for fertilizing capacity [9].

The mitochondrial DNA genes lack introns and intergenic non-coding nucleotides [10, 11]. The mtDNA repair system does exist and not enough to overcome the oxidative damage continued by the mitochondrial genome as a result of the closeness to the respiratory chain complexes in the mitochondrial membrane (MIM) which generate ROS. Therefore, the range of mtDNA mutation is considerably high (10–17-fold higher) compared to nuclear DNA [10, 12]. The mtDNA mutations can deteriorate the energy creation and the organism’s health, as proteins encoded in the mtDNA are the basics of the OXPHOS [13]. Mutations within mtDNA may cause the beginning of mitochondrial diseases, which are a major group of genetic disorders [14, 15].

The human sperm mitochondrial ultrastructure defects appear to link with low sperm motility [16]. Earlier work has discovered that mtDNA variations which impact cellular homeostasis may give rise to reduce fertility [17]. Also, Jodar et al. [18] advised that the alteration in the specific mRNA’s transcripts in sperm cells of Asthenozoospermic patients arise along with mitochondrial protein nuclear-encoded transcripts. Numerous studies have itemized links between mitochondrial genetic variation and deleterious male reproductive outcomes. They suggest that the progress of compensatory mechanisms is partially active in offsetting the male-harming mtDNA mutations effects. Additionally, there seems to be a sex bias developing in the reporting of such effects, by many studies proposing that different mtDNA variants affect constituents of male fertility [19–21].

The SNPs happened when errors occur (substitution, insertion, and deletion), and they are prominent sources of variation in the human genome and aid as excellent genetic markers for constructing high genetic maps and to carry out association studies related to diseases. Most SNPs are situated in non-coding regions of the genome and have no direct identified impact on the phenotype of an individual, but their character till now stays elusive, and liable on where SNPs happen; it might have unlike consequences at the phenotypic level [22]. Many studies reported that there is an association between the presence of mitochondrial DNA (mtDNA) variation and infertility in the Tunisian [23], Jordanian [24], and Iranian [25] populations.

The current study is considered the first study in the Egyptian population concerning mitochondrial DNA (mtDNA) variation, especially in asthenozoospermic patients. The work was planned to evaluate the relation between the occurrences of the mtDNA mutations (SNPs) in asthenozoospermic patient semen in the ND1, ND2, and ATPase6 genes and the spermatozoa motility.

## Methods

### Sample collection and semenological analysis

Human semen samples were obtained from patients with an age ranging from 21 to 45 years at the Adam International Hospital for Reproductive Medicine in Giza, Egypt. Semen samples were collected from 75 patients which included 25 with normal motility value (normozoospermic group) and 50 with pathogenic (asthenzoospermia group) and processed in the laboratory within an hour of ejaculation. Semen was collected by masturbation into sterile disposable containers after 3–5 days of sexual abstinence and allowed to liquefy for 30–60 min. Then, routine seminal analyses were performed according to the World Health Organization (WHO) guideline [26]. Ethical approval was obtained from the Ethics Committees of the National Research Centre (Approval No. 16/455), and informed consent was obtained from all subjects.

The liquefaction characteristics of the semen are examined 20 min after ejaculation and recorded. All samples were assessed according to World Health Organization (WHO) guidelines (2010). The following variables were taken into consideration: ejaculate volume (ml), sperm concentration (10^6^/ml), total sperm number (10^6^/ejaculate), progressive motility (%), morphology (% abnormal forms), and leukocyte concentration (10^6^/ml). Semen samples with leukocytospermia and/or increased viscosity were excluded from this study. A sperm viability test was carried out to differentiate cell death from immotility by staining with eosin Y 0.5% in saline solution. These samples were divided into two groups on the basis of their progressive motility, using the WHO 2010 5th percentile as the cutoff: the asthenozoospermic group comprises samples with progressive motility < 32%, and the normozoospermic group comprises samples with progressive motility ≥ 32%.

### Measurement of lipid peroxidation

Malondialdehyde levels were determined by the malondialdehyde (MDA)-thiobarbituric acid (TBA) test which is the colorimetric reaction of MDA and TBA in acid solution. TBA reacted with MDA, a secondary product from lipid peroxidation, which generated an adduct of red color, which was detected spectrophotometrically [27].

### Measurement of fructose in semen

The fructose measurement may help in assessing the diagnosis and the management of male infertility. Fructose seminal concentration has negative correlations with sperm concentration and motility [28]. Fructose was measured according to the protocol of Foreman et al. [29], which forms a pink color when heated with resorcinol in the presence of hydrochloric acid, which can be directly measured photometrically.

### Measurement of total antioxidant capacity

The determination of the total antioxidant capacity is performed by the reaction of antioxidants in the sample with a defined amount of exogenously provide hydrogen peroxide (H_2_O_2_) according to the protocol of Koracevic et al. [30]. The antioxidants in the sample eliminate a certain amount of the provided hydrogen peroxide. The residual H_2_O_2_ is determined calorimetrically by an enzymatic reaction which involves the conversion of 3,5,dichloro-2-hydroxybenzensulphonate to a colored product according to the manufacturer’s instruction.

### Extraction of total DNA and quantification

The total DNA from the sperm cells was isolated using the QIAamp DNA Mini kit (QIAGEN) as per the manufacturer’s instruction: https://www.qiagen.com. Three microliters of the extracted DNA and 2 μl of DNA gel loading dye (6×) were mixed then loaded in a 1.5% agarose gel to determine the integrity of DNA, and electrophoresis was carried out at 100 V/cm for 30 min. The photograph was examined below UV light by a gel documentation imaging system (Bio-Rad, USA). The extracted DNA was quantified using the NanoDrop Lite Spectrophotometer (Thermo Scientific).

### Polymerase chain reaction analysis

The ND1, ND2, and ATPase 6 mitochondrial genes are amplified using 2 sets of designed PCR primers, which were located in the flanking regions of each gene as illustrated in Table 1. For facilitating the work, a master mix was usually prepared first, and 18 μl from this mix was dispersed in the 0.2-ml Eppendorf tube and 2 μl from the template DNA is added. The master mix formula was as in Table 2. The conditions of PCR amplification were as follows: a denaturation step at 94 °C for 5 min followed by 35 cycles at 94 °C for 1 min, annealing for 1 min (the annealing temperature varied depending on the primer size, length of amplified fragment, “GC” content, and the structure of DNA fragment (Table 1)) and 72 °C for 1 min and then a final extension at 72 °C for 5 min and stop at 4 °C [31].

**Table 1:** Primer pairs used to amplify study genes

Primer name	Genomic location (bp)	Sequence (5′-3′)	GC content (%)	Annealing temp.	Product size (bp)
ND1 F ND1 R	3245-3264 4370-4351	CCCCGGTAATCGCATAAAACT TTTTGGATTCTCAGGGATGG	45 45	66oC 66oC	1125
ND2 F ND2 R	4382-4402 5565-5545	CCTATCACACCCCATCCTAAA TGCAACTTACTGAGGGCTTTG	47 47	69oC 69oC	1183
ATPase6 F ATPase6 R	8531-8552 9185-9206	AAC GAA AAT CTG TTC GCT TCA ATG TGT TG T CGT GCA GGT GA	47 47	69oC 69oC	675

*F* forward primer, *R* reverse primer, *bp* base pair position related to the reference human mitochondrial sequence (accession number NC_012920.1)

**Table 2:** The values of a master mix components

Component	Volume (μl)	Final concentration
DNA sample template	2.0	50 ng
10× PCR reaction buffer	2.0	1×
Forward primer	1.5	1 pmol
Reverse primer	1.5	1 pmol
dNTPs	2.0	200 μM
TaqDNA polymerase	X	1 U
Sterile double-distilled water	X	Up to 20 μl

### Gene sequence analysis

The sequencing was done by Macrogen Incorporation (Seoul, Korea) after the purification procedure was done by using a Thermo Scientific purification kit of PCR product. The variants were imported into the BioEdit sequence alignment editor version 7.2.1 and aligned with the reference human mtDNA where the mutations at various points were identified and recorded.

### Polymorphisms screening

The DNA sequence variations were determined by alignment of study gene sequence against database sequences using nBLAST tool https://blast.ncbi.nlm.nih.gov/Blast.cgi. These sequences were compared to the reference sequence (accession number: NC_012920.1). Mutation type (synonymous or non-synonymous) sequence translated to amino acid was determined by the Expasy program https://web.expasy.org/translate/. The single nucleotide polymorphisms (SNPs) were detected within 15 samples, 7 of normozoospermia and 8 for asthenozoospermia by the mitomap software http://www.mitomap.org to differentiate between old mutation and new (novel) SNPs. Analysis of the effect of the non-synonymous mutations was done by the SIFT and PROVEAN software. SIFT relies on sequence homology in order to sort intolerant from tolerant mutations http://sift.jcvi.org/. Additionally, the PROVEAN software is a sequence-based predictor that estimates whether a protein sequence variation affects protein function or not http://provean.jcvi.org.

### Statistical analysis

The Statistical Package for the Social Sciences (SPSS) 20.0 (SPSS, Inc., Chicago, USA) was used for the statistical analysis. Continuous variables for each group of patients were expressed as mean ± standard error of the mean. The statistical analysis was conducted by comparing the distribution of each variable of the two study groups using Student’s *t* test, as appropriate. The correlations among the variables were evaluated by calculating Pearson’s correlation coefficient. For statistical purposes, *p* ≤ 0.05 was considered as significant.

## Results

### Semen physical character

Table 3 illustrates the basic semen characters, sperm concentration, morphological parameters, total motility, and progressive motility percentages of normozoospermic and asthenozoospermia infertile men. The result of the present investigation revealed that the percentages of spermatozoa total and progressive motility were significantly low (*p* ≤ 0.001) in asthenozoospermia infertile men (21.45 ± 1.81; 5.27 ± 0.9, respectively) as compared to those of normozoospermic men (53.13 ± 1.91; 12.31 ± 1.10, respectively). The sperm count was significantly decreased (*p* ≤ 0.001) in the infertile asthenozoospermic men (34.27 ± 8.82) than those of the normozoospermic men (85.83 ± 14.35). Differences between the mean percentages of sperm morphology, teratozoospermia index, and acrosome index of fertile and infertile men (91.3 ± 1.0, 1.4 ± 0.04, and 57.8 ± 3.8 versus 95.3 ± 0.6, 1.6 ± 0.06, and 46.7 ± 3.4, respectively) were significant at *p* ≤ 0.05. While no significant difference in the sperm deformative index (SDI) of the two groups.

**Table 3:** physical parameters of normozoospermic and asthenozoospermic men

Parameters	Normozoospermia	Asthenozoospermia
Total motility (%)	53.13 ± 1.91**	21.45 ± 1.81
Progressive motility (%)	12.31 ± 1.10**	5.27 ± 0.9
Sperm count (10^6^)	85.83 ± 14.35**	34.27 ± 8.82
Sperm morphology (%)	91.3 ± 1.0	95.3 ± 0.6 ± 0.6*
TZI (%)	1.4 ± 0.04	1.64 ± 0.06*
Acrosome index (%)	57.8 ± 3.8*	46.7 ± 3.4
SDI	1.71 ± 0.08	1.85 ± 0.09

Values represent as mean ± SEM, *p ≤ 0.05, **p ≤ 0.01

*TZI* teratozoospermia index, *SDI* sperm deformative index

### Semen biochemical results

Table 4 represents the malondialdehyde, total antioxidant activity, and fructose levels in normozoospermia and asthenozoospermia samples. The level of malondialdehyde (MDA) in the seminal plasma of the asthenozoospermic men was significantly higher (*p* ≤ 0.01) than that in healthy normozoospermic fertile men. The seminal total antioxidant capacity test revealed significantly lower activity in asthenozoospermia (*p* ≤ 0.05) compared to normozoospermia, whereas the results of seminal plasma fructose indicated there was no significant difference in the fructose level of normozoospermia and asthenozoospermia cases.

**Table 4:** Biochemical parameters of seminal plasma

Parameters	Normozoospermia	Asthenozoospermia
MDA (nmol/l)	6.02 ± 0.51	9.89 ± 0.68**
TAC (mM/l)	1.81 ± 0.08*	1.56 ± 0.07
Fructose (mg/dl)	241.00 ± 9.57	225.00 ± 9.47

Values represent as mean ± SEM, *p ≤ 0.05, **p ≤ 0.01

*MDA* malondialdehyde, *TCA* total antioxidant capacity

**Fig. 1:**
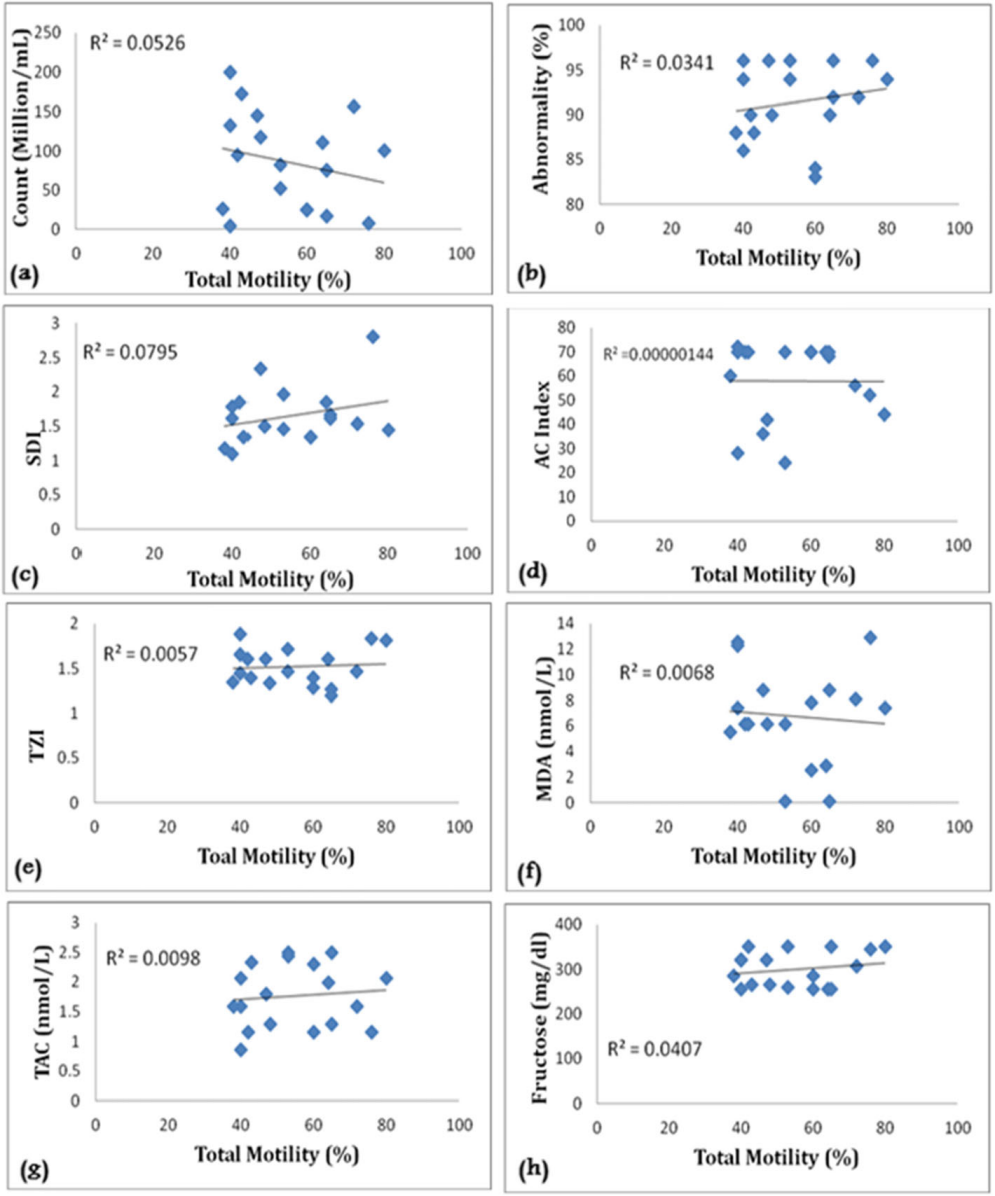
Correlation coefficient between total motility in the normozoospermia group. **a** Sperm count. **b** Abnormality (%). **c** Sperm deformative index. **d** Acrosome index. **e** Teratozoospermia index. **f** Malondialdehyde. **g** Total antioxidant capacity. **h** Fructose level

**Fig. 2:**
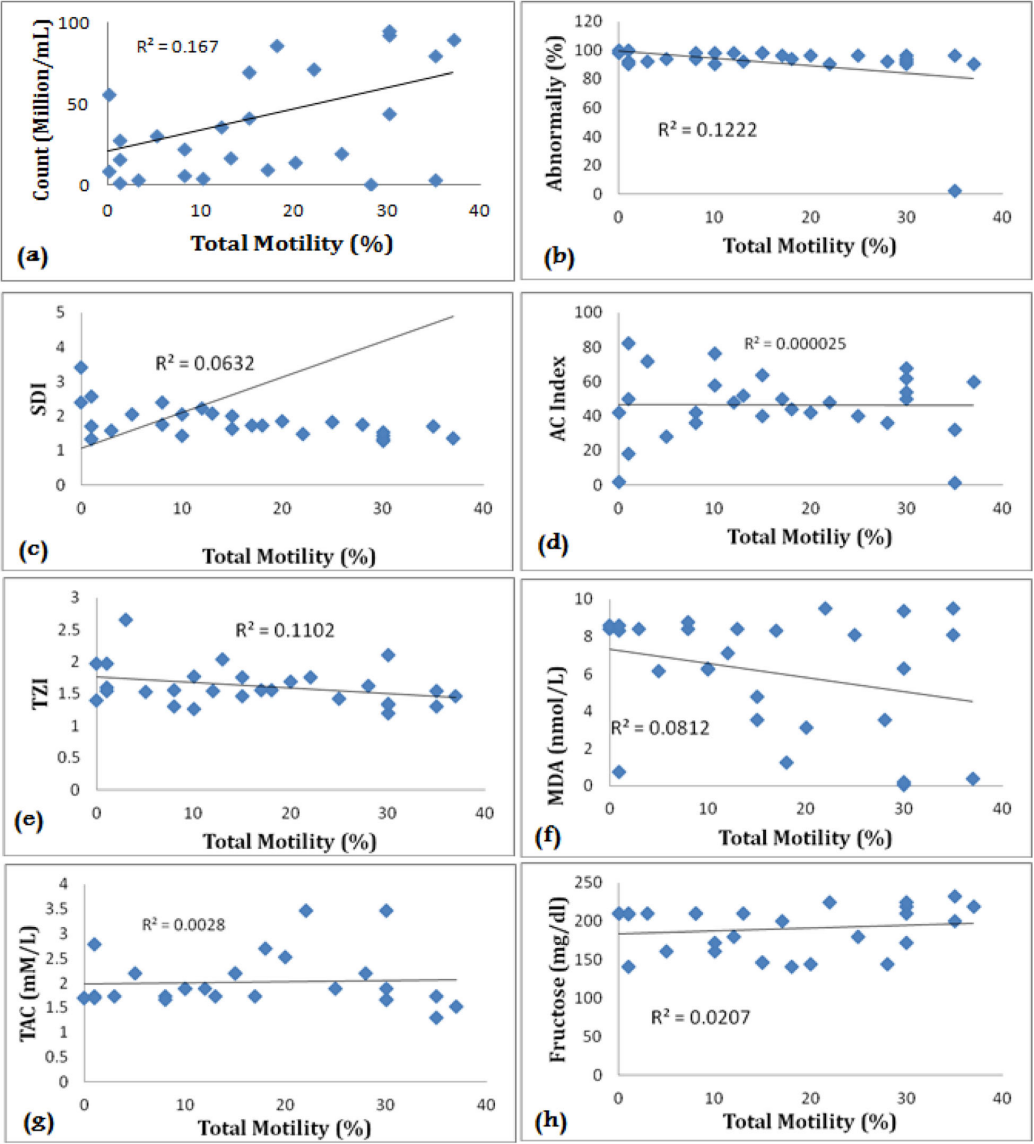
Correlation coefficient between total motility. **a** Sperm count. **b** Abnormality (%). **c** Sperm deformative index. **d** Acrosome index. **e** Teratozoospermia index. **f** Malondialdehyde. **g** Total antioxidant capacity. **h** Fructose level

Our findings showed that there was a negative correlation between motility with MDA levels (*r* = − 0.082), sperm concentration (*r* = − 0.229), and acrosome index (*r* = − 0.0012), while this correlation was positive between motility and total antioxidant capacity (*r* = 0.098), seminal fructose level (*r* = 0.201), and morphology (*r* = 0.184) in the Normozoospermia group as shown in Fig. 1. While there was a negative correlation between motility with MDA levels (*r* = − 0. 282), sperm morphology (*r* = − 0. 349), TZI (*r* = − 0.291), and acrosome index (*r* = − 0. 005) in the asthenozoospermia group. The correlation between motility and sperm concentration (*r* = 0. 0.408), total antioxidant capacity (*r* = 0. 052), and seminal fructose level (*r* = 0. 144) in the asthenozoospermia group was significantly positive (*p* ≤ 0.05) as illustrated in Fig. 2.

### Molecular results

**Fig. 3:**
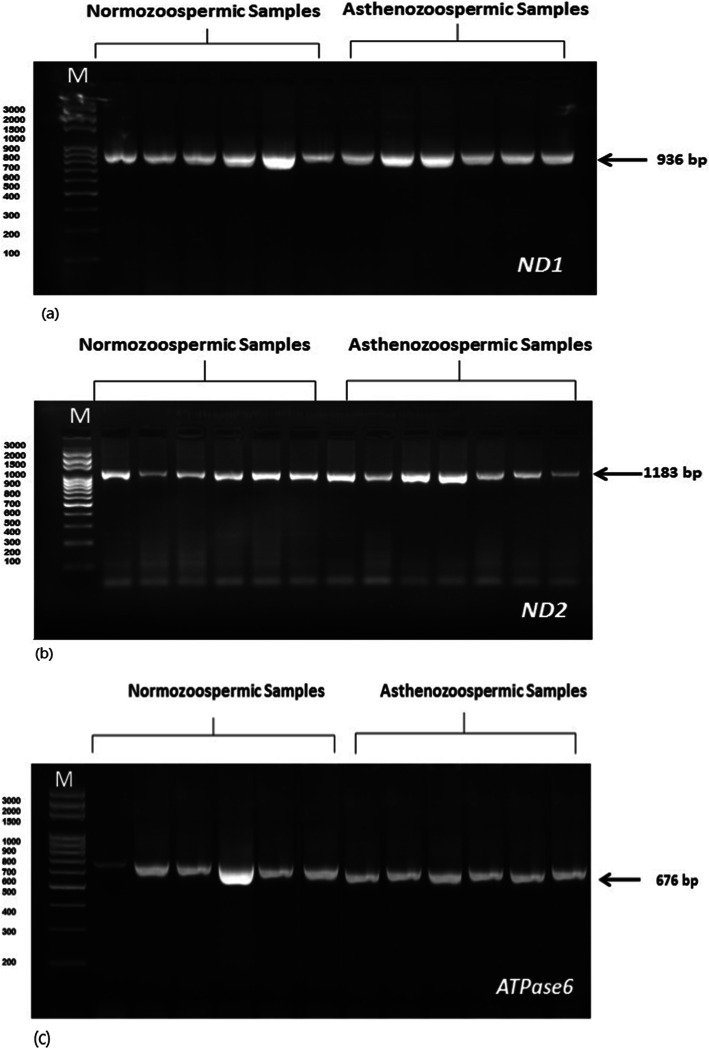
PCR products. Lane M: lambda DNA marker digested with restriction enzymes; lanes 1–6: normozoospermia samples; lanes 7–12: asthenozoospermia samples. **a** ND1 gene. **b** ND2 gene. **c** ATPase6 gene

Amplification of a fragment from the *MT-ND1*, *MT-ND2*, and *MT-ATPase* genes of human semen samples formed 936, 1183, and 676 bp PCR product, respectively (Fig. 3). The sequence analysis of *MT-ND1*, *MT-ND2*, and *MT-ATPase* genes in normozoospermia and asthenozoospermia samples revealed the presence of several mutations in different nucleotide positions in genes as illustrated in Table 5. Concisely, 31 diverse nucleotide substitutions were recognized, and most mutations are transitional mutations.

**Table 5:** The mtDNA mutations identified in the ND1, ND2, and ATPase6 genes

Nucleotide position	Nucleotide change	Amino acid change	Mutation in Normo.	Mutation in Astheno.	Frequency (%)	Mitomap (Y)
ND1 gene						
3396	T–C	Silent		1	6.6	Y
3398	T–C	M–T		1	6.6	X
3423	T–C	Silent		1	6.6	X
3594	C–T	Silent		2	13.3	Y
3693	G–A	Silent		**1**	6.6	Y
3705	G–A	Silent		**2**	13.3	Y
3821	T–C	L–P		**1**	6.6	Y
4048	G–A	D–N		**1**	6.6	Y
4104	A–G	Silent	**1**	**2**	20.0	Y
4158	A–G	Silent	**1**		6.6	Y
4169	Ins. T	L–P	**1**	**1**	13.3	Y
4216	T–C	Y–H	**1**		6.6	Y
ND2 gene						
4613	Del A	Silent	**1**	**1**	13.3	X
4767	A–G	M–V	**1**	**6**	46.6	Y
4769	A–G	Silent	**1**	**1**	13.3	Y
4856	T–C	Silent		**1**	6.6	Y
4940	C–T	Silent		**1**	6.6	Y
4958	A–G	Silent		**1**	13.3	Y
4991	G–A	Silent	**2**	**1**	20.0	Y
5004	T–C	Silent		**1**	6.6	Y
5027	C–T	Silent	**1**		6.6	Y
5111	C–T	Silent		**1**	6.6	Y
5147	G–A	Silent	**1**	**3**	26.6	Y
5320	C–T	T–I	**1**	**1**	13.3	Y
5331	C–A	P–H	**1**		6.6	Y
5351	A–G	Silent	**2**	**1**	20.0	Y
ATPase 6 gene						
8697	G–A	Silent	**1**		6.6	Y
8701	A–G	T–A	1	**6**	46.6	Y
8860	A–G	T–A	1	**6**	46.6	Y
9075	C–T	Silent	1	**2**	13.3	Y
9148	T–C	Silent		**1**	6.6	Y
Total mutation numbers = 31						

*Y* the mutation proven by www.mitomap.org, *X* novel mutation

**Fig. 4:**
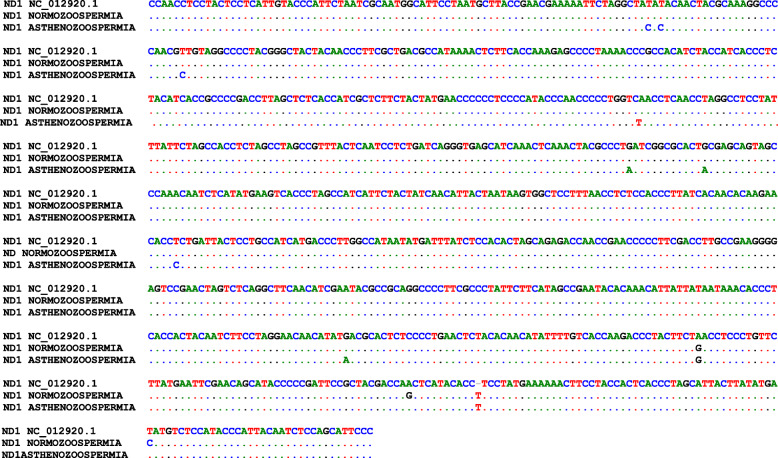
The sequence alignment of the ND1 gene of normozoospermia and asthenozoospermia samples with reference sequence obtained from the NCBI database (accession number: NC_012920.1) by the BioEdit software

Alignment of the sequence of the genes discovered the incidence of twelve point mutations in MT-ND1, one mutation was insertion and the others were of transition type (Fig. 4). The normozoospermia samples yielded four SNPs which were two synonymous mutations at nucleotides A4104G and A4158G, besides two non-synonymous mutations at positions *insertion* (T) 4169 and T4216C. Sequence analysis of the ND1 gene in asthenozoospermia samples yielded ten SNPs which were detected, six of which were synonymous mutations at nucleotides T3396C, T3423C, C3594T, G3693A, G3705A, and A4104G, and the others were non-synonymous mutation at positions T3398C, T3821C, G4048A, and insertion (T) 4169.

**Fig. 5:**
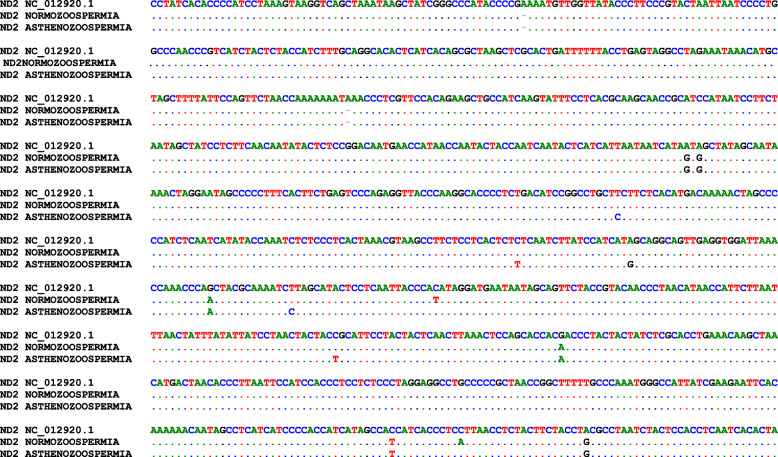
The sequence alignment of the *ND2* gene of normozoospermia and asthenozoospermia samples with reference sequence obtained from the NCBI database (accession number: NC_012920.1) by the BioEdit software

In the ND2 gene, fourteen point mutations were characterized (Fig. 5). Sequence analysis of the ND2 gene in mormozoospermia samples yielded nine base substitutions; six of them were synonymous; five are transition A4769G, G4991A, C5027T, G5147A, and A5351G; and one SNP of deletion type at nucleotide number deletion (A) 4613. The other three mutations were non-synonymous, two are transition at nucleotide positions A4767G and C5320T, and the latter is of transversion type at nt C5331A. Twelve SNPs were detected in the asthenozoospermic group; ten base substitutions were synonymous while two were non-synonymous at nucleotide numbers A4767G and C5320T. The comparison applied by BioEdit revealed that the seven base substitutions located at nucleotides (nts) deletion (A) 4613, A4767G, A4769G, G4991A, G5147A, C5320T, and A5351G are sharing between the two groups. One SNP of deletion type at nucleotide number 4613 appeared in the two groups.

**Fig. 6:**
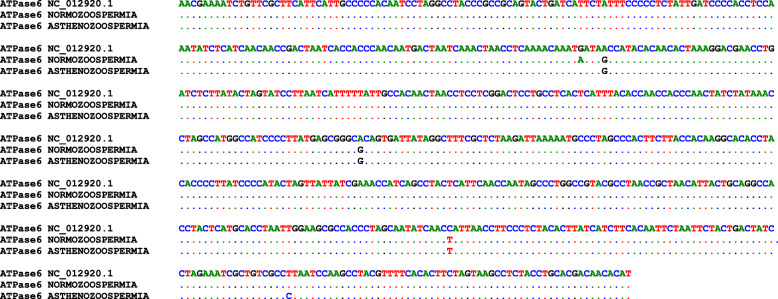
The sequence alignment of ATPase 6 gene of normozoospermia and asthenozoospermia samples with reference sequence obtained from the NCBI database (accession number: NC_012920.1) by the BioEdit software

In addition, in the MT-ATPase6 gene, five mutations were found at different nucleotide positions in the normozoospermia and asthenozoospermia samples after alignment (Fig. 6). Three transition mutations were identified in both two groups, A8701G, A8860G, and C9075T, and G8697A appeared in normozoospermia and T9148C in asthenozoospermia.

The transition mutations involve the substitution of either a purine for a purine or a pyrimidine for a pyrimidine (i.e., A>G, G>A, C>T, or T>C). Out of the thirty-one (31) nucleotide changes observed, only ten (10) mutations led to the amino acid change in which four have a deleterious effect, four are benign, and two mutations have conflicting results about effectiveness. T3398C, T3821C, G4048A, Ins T4169, T4216C, A4767G, C5320T, C5331A, A8701G, and A8860G caused a change from methionine to threonine, leucine to proline, aspartic acid to asparagine, leucine to proline, tyrosine to histidine, methionine to valine, threonine to isoleucine, proline to histidine, threonine to alanine, and threonine to alanine, respectively, as shown in Table 6.

**Table 6:** Predicted amino acid substitution effects in tested genes

Variant	PROVEAN score	Prediction (cutoff = − 2.5)
**ND1**		
**M15T**	**− 5.188**	**Deleterious**
**L156P**	**− 3.248**	**Deleterious**
**D232N**	**1.312**	**Neutral**
*L273P*	*− 4.407*	*Deleterious*
Y288H	− 0.759	Neutral
**ND2**		
***M33V***	***− 0.092***	***Neutral***
***T217I***	***2.952***	***Neutral***
**L221I**	**− 1.829**	**Neutral**
**ATPase 6**		
**T3A**	***− 0.830***	*Neutral*
**T56A**	***− 4.013***	*Deleterious*

Italic amino acid indicates a mutual change in the normozoospermic and asthenozoospermic groups. Bold amino acid indicates a mutual change in the asthenozoospermic group only

All SNPs were proven by *mitomap* except three SNPs which were novel mutations; T3398C was a non-synonymous mutation in which amino acid changed from methionine to threonine and had a deleterious effect on protein function. This mutation appeared only in asthenozoospermic samples which might cause low motility and infertility. The second mutation at position T3423C also appeared in asthenozoospermia only but was a synonymous mutation. Deletion (A) at nucleotide position 4613 was a synonymous mutation and occurred in both groups.

## Discussion

Male infertility is a medical and psychosocial global problem, and it accounts for approximately 40% of infertility in couples. Infertility itself affects about 7% of all couples attempting pregnancy [1]. Asthenozoospermia is a multifactor syndrome that affects approximately half of males with infertility. It could possibly be caused by defects in spermatozoa tail development or by energy-producing machinery defects that are essential to drive motility [32].

Oxidative stress has been recognized as an intermediary of male infertility through affecting sperm dysfunction. Although ROS minor amounts are necessary for sperm function, unbalanced levels can adversely impact the spermatozoa quality and their fertilizing capacity [33]. The data of the current inquiry noticed that asthenozoospermic infertile men were categorized with low total and progressive spermatozoa motility and a high percentage of sperm morphology and teratozoospermia index compared to normozoospermic men. In addition, there was a negative correlation between seminal plasma malondialdehyde (MDA) levels and sperm motility. This suggests that infertile patients undergo oxidative stress-induced lipid peroxidation, where malondialdehyde is an important marker for oxidative stress. A rise in MDA could be due to the increased generation of reactive oxygen species due to the excessive oxidative damage generated in infertile men. This oxygen species in turn can oxidize the lipids in the sperm membranes causing alterations in the sperm that may reduce fertility by affecting sperm motility [34]. Also, the resulting oxygen species usually manifests in a range of adverse sequelae that drive germ cell dysfunction and terminate their apoptotic demise [35]. This result is in accordance with the study of Masroor et al. [36]; they observed that the seminal plasma level of MDA was higher in asthenozoospermic males than in normozoospermic males. Regarding the relation between seminal MDA level and spermatozoa parameters in infertile men, Al-azzawie et al. [37] revealed that higher seminal MDA levels were adversely related to progressive and non-progressive sperm motility and normal sperm morphology, whereas they were positively allied with immotile sperm. Nowicka-Bauer et al. [38] established that sperm dysfunction leading to asthenozoospermia, faulty mitochondria, and an associated reduction in the energy production that is vital to support normal movement have been identified as a common etiology*.* The assessment of seminal total antioxidant has been recommended as an appreciated tool to develop the estimation of sperm reproductive capacity and functional competence in infertile men. With this in mind, the result of total antioxidant capacity (TAC) analysis in the current study revealed significantly lower activity in asthenozoospermic. Our result was similar to Salimi et al. [39], and Hosen et al. [40] studies established that the total antioxidant capacity value in infertile men was lower than that in fertile men. In addition, Riaz et al .[41] and Ajina et al. [42] have shown the same results in asthenozoospermic men mentioning that low seminal total antioxidant capacity may be associated with a high concentration of ROS creation and might have a major role in the etiology of sperm abnormality. Fructose acts as an energy donor to the spermatozoa that break it down selectively and change it into energy. Fructose is reported to play an imperative role in sperm motility and concentration. Fructose is the main carbohydrate created in seminal plasma and looks crucial for sperm motility [28]. The result of seminal plasma fructose assessment indicates that the level of fructose concentration in asthenozoospermic patients was non-significantly decreased than in normozoospermic. Our results confirm those reported previously by Thi Trang et al. [28] that fructose seminal concentration has negative connections with sperm concentration and motility.

Sperm motility is a fundamental requirement to ensure male fertility. Correspondingly, a number of studies focused on the mitochondria, indicating their key role in cellular homeostasis and sperm motility [43]. Recent experiments in sperm physiology are concerning with the mitochondria as sperm health and fertility biomarker [44, 45]. In addition, the mitochondrial sperm dysfunction is equally concerned in the pathogenesis of seminal oxidative stress that can be answerable in various cases of male idiopathic [46]. It has been confirmed that mtDNA base substitutions can really impact semen quality and motility [47]. Sperm mtDNA variations result in functionless proteins disturbing sperm motility which is strongly dependent on ATP biosynthesis which is carried out by the mitochondrial OXPHOS system [48]. The mtDNA rearrangement has been stated in asthenozoospermic patients [49]. The mtDNA variation has been established in the framework of various multifactorial diseases [23, 50, 51]. While mitochondrial mutations and their correlation to male infertility have been broadly investigated, there are still contradictions in data. Whereas numerous studies have informed association among male infertility have been broadly investigated, there are still contradictions in data. Whereas several studies have informed the association between male infertility and mtDNA mutations in certain genes [52–54], others did not [55, 56]. The current study investigated the mtDNA mutations of asthenozoospermic men, and it provides the baseline data on mtDNA mutations among infertile and fertile Egyptian men for the first time. The results revealed that five non-synonymous SNPs (nsSNPs) located at nucleotides T3398C, T3821C, G4048A, T4169TT, and T4216C in the ND1 gene that produced amino acid convert from methionine to threonine, leucine to proline, aspartic to asparagine, leucine to proline, and tyrosine to histidine, respectively. Secondary structure prediction of the proteins of T3398C and T3821C showed a detrimental change in protein function, so this mutation may cause sperm motility decline and male infertility. Mughal et al. [57] settled that the mtDNA mutations had a significant influence on sperm quality causing infertility by affecting various sperm motility parameters. Zhang et al. [58] reported that due to the low occurrence frequency and limited sample size, m C3398T showed a decreased risk of asthenozoospermia. The mitochondrial transition mutation T4216C was found only in normozoospermic men with a percentage of 14.2%. While Khan et al. [59] and Stenson et al. [60] confirmed that there was a genetic mutation in the base substitutions that do change in the SNP T4216C position in fertile and subfertile men. It was observed in asthenozoospermic man and change synonym in tyrosine codon third position converting it from TAT to TAC. The non-synonymous (nsSNPs) are the greatest mutual genetic variation type. They can affect the protein function leading to an impact on human health [61].

Additionally, the present results displayed that subfertile patients had twelve mutual SNPs in the ND2 gene. Ten SNPs were synonymous (nine transition mutations, T4856C, C4940T, A4958G, T5004C, C5111T, A4769G, G4991A, G5147A, and A5351G) plus one deletion type at nucleotide (A) 4613. The other two mutations were non-synonymous at positions A4767G and C5320T. In addition, synonymous mutations (C7024T and C7891T) in COXI and COXII were recognized as T polymorphic sites in men with low sperm mobility [62]. The synonymous mutations found by Harris et al. [63] also showed 58% of subfertile men have reduced relative codon usage and 33% with higher and 8.33% did not show any transformation in their relative codon usage. Likewise, additional synonymous mutations in the subfertile group were connected with reduced relative codon usage. All these findings recommended that synonymous codons with low frequency rate are more recurrent in subfertile men. The non-synonymous single nucleotide polymorphisms (nsSNPs) are the greatest mutual type of genetic variation. They can affect the protein function leading to an impact on human health compared with SNPs in other regions of the genome [61].

Zhang et al. [58] detected the mitochondrial mutations T4856C, C4940T, G4991A, and G5147A in the MT-ND2 gene of the total fertilization failure (TFF) group, but there were no significant differences in the frequencies of point heteroplasmic variants between the TFF and control. Also, no significant variation in the frequencies of mtDNA haplogroup D or G among the IVF fertilization failure and the normal groups. They conclude that no significant difference in the Han Chinese population but might affect other populations. Barbhuiya et al. [54] and Bimah [64] found that the non-synonymous transition mutation A>G at nt 4769 was noticed in all the cases of both control and infertile groups.

Associating the present results with the earlier studies, one can find worthy evidence for applying the ATPase 6 gene in differentiating between fertile and infertile men [65]. The sequence of MT-ATPase 6 gene revealed a high frequency of nucleotide changes in asthenozoospermic men. Significant non-synonymous nucleotide mutations (nsSNPs) A8701G and A8860G were identified in the asthenozoospermic group with 75% changed from threonine to alanine, while synonymous (sSNPs) G8697A and C9075T were found in the normozoospermic group with 14%, while the T9148C was observed in asthenozoospermic in leucine codon by 12.5%.

These findings were agreed with that of Holyoake et al.’s [66] investigation on samples from New Zealand in sharing the non-synonymous sites G8860A. Our results agreed also with that of [53] in characterizing the non-synonymous sites A8701G and G8860A in oligoasthenozoospermic men. These mutations changed the amino acid from threonine to alanine and may affect sperm motility. Also, our results are comparable to the results of Kumar and Sangeetha [53] asserting that A8860G nucleotide substitution was present in infertile men with 91% and in control with 47%. In addition, many authors found that base substitution reversed to our result at nucleotide 8860 (G to A) within ATPase 6, changing from an alanine to a threonine [55, 67]. Moreover, Barbhuiya et al. [54] observed the mutations A4769G, A8702G, and A8860G in both fertile and infertile males. These notes suggest that these mutations might be prevalent mutations in the general population and not allied with male infertility. Bimah et al. [64] confirmed that the frequencies of occurrence of the mutations at nts 8860 and 8701 were a hundred percent (100%) in both the normozoospermic and abnormospermic subjects. Elsanousi et al. [65] sequenced the ATPase subunit 6 mitochondrial genes (ATPase 6) for infertile and normal Saudi men to identify the SNPs associated with male infertility. Seven non-synonymous substitutions were obtained with 4 novel sites (C8684T, G8860A, C8876T, and G9055A) in asthenozoospermic men. It could be recognized that there was a link between ATPase 6 substitutions and male sterility.

Mitochondrial genome nucleotide substitutions have a relation with a variety of metabolic pathologies; nevertheless, investigation of the outcome of definite mtDNA genotypes is continuous. mtDNA alterations have specific importance for reproductive characters; subsequently, they are predicted to have deep impacts on male exact procedures because of the exact maternal inheritance of mtDNA [21].

Contrary to the aforesaid evidence, Pereira et al. [6] analyzed the complete mtDNA of asthenozoospermic patients and whole mitogenomes of teratoasthenozoospermic males and confirmed no association between male infertility and mitogenomes. Similarly, Palanichamy and Zhang [56] supposed that no relationship between the mtDNA substitutions and infertility in males. These authors considered the published data are the misleading application of the mitochondrial polymorphisms in male sterility diagnosis.

The malfunctioning protein subunits due to mutated mtDNA, when collected with nuclear-encoded subunits, lead to respiratory enzyme anomaly [68]. Spermatozoa with mutant mtDNA are producing less efficient ATP and generate extra ROS that may impair the mitochondria and mtDNA causing definitive energy crisis, motility, and fertility decline [9, 69].

## Conclusion

In summary, the first study on the Egyptian population demonstrated that the incidences of the mtDNAs with the ND1, ND2, and ATPase6 genes are positively associated with spermatozoa motility and fertility. Although the small sample size and focusing on the asthenozoospermic Egyptian population, it was hard to confirm the effect of mitochondrial mutations in male infertility. The results showed scientific indications evidenced that there is a connection between mitochondrial mutations and infertility in some tested cases. Additional studies would comprise a more patient number, genes, and SNPs which will eventually aid us to realize the individual genetic factors influence the progress of male infertility.

## Data Availability

The data and materials are available.

## Abbreviations

ATP: Adenine triphosphate
ATPase6: ATP synthase membrane subunit 6 gene
bp: Base pair
dATP: Deoxyadenosine triphosohate
ETC: Electron transport chain
G: Guanine or guanosine; glycine
gm: Grams
h: Hours
H_2_O_2_: Hydrogen peroxide
Ma: Milliampere
MDA: Malondialdehyde
MIM: Mitochondrial membrane
min: Minutes
mRNA: Messenger ribonucleic acid
mtDNA: Mitochondrial deoxyribonucleic acid
NADPH: Nicotinamide adenine dinucleotide phosphate
NCBI: National Centre of Biotechnology Information
ND1: NADH dehydrogenase 1
ND2: NADH dehydrogenase 2
nDNA: Nuclear deoxyribonucleic acid
ng: Nanogram
nmol: Nanomole
np: Nucleotide position
nt: Nucleotide
OAT: Oligoasthenoteratozoospermia
OS: Oxidative stress
OXPHOS: Oxidative phosphorylation system
PCR: Polymerase chain reaction
PR: Progressively
PUFAs: Polyunsaturated fatty acids
RNA: Ribonucleic acid
ROS: Reactive oxygen species
rRNAs: Ribosomal ribonucleic acid
SDI: Sperm deformative index
SEM: Slandered error of the mean
SNPs: Single nucleotide polymorphisms
sSNPs: Synonymous single nucleotide polymorphisms
T: Thymine or thymidine; threonine
TAC: Total antioxidant capacity
Taq DNA: Thermus aquaticus DNA
TBA: Thiobarbituric acid
Tm: Melting temperature
tRNAs: Transfer ribonucleic acid
U: Unit
UV: Ultraviolet
